# Supplementary material for: Psychological and Physical Changes Caused by COVID-19 Pandemic in Elementary and Junior High School Teachers: A Cross-Sectional Study
Source: Int J Environ Res Public Health. 2022 Jun 21;19(13):7568. doi: 10.3390/ijerph19137568 (PMC9265812; doi:10.3390/ijerph19137568)
Supplement: Supplementary file 1 [file ijerph-19-07568-s001.zip › ijerph-1760610-supplementary.pdf]

COVID-19 questionnaire survey

School Type □ Elementary School □ Junior high school

Sex □Male □ Female

Age _________

A question about changes in subjective symptoms before and after the COVID-19 pandemic. Please circle the relevant part.				
Do you have changes in psychological conditions before and after school closures due to COVID-19 ?		Improved	No Changed	Deteriorated
Q1-1	Feeling high			
Q1-2	Irritation			
Q1-3	Anger			
Q1-4	Indignation			
Q1-5	Anxiety			
Q1-6	Chagrin			
Q1-7	Impotent feeling			
Q1-8	Depression			
Q1-9	No sense of reality			
Q1-10	No sense of time			
Q1-11	Emotions are paralyzed			
Q1-12	I cannot concentrate on work at all			
Q1-13	No longer wanting to engage with others			
Do you have changes in physical symptoms before and after school closures due to COVID-19 ?		Improved	No Changed	Deteriorated
Q2-1	Insomnia/nightmares			
Q2-2	Palpitations			
Q2-3	Standing dizziness			
Q2-4	Sweating			
Q2-5	Dyspnea			
Q2-6	Digestive symptoms			
Did COVID-19 impact your work?		Improved	No Changed	Deteriorated
Q3-1	Excessively immersed in the work			
Q3-2	Lowering of the thinking ability			
Q3-3	Lowering of the concentration ability			
Q3-4	Lowering of the work efficiency			
What is your stress causes for teachers associated with COVID-19 infection control ? Please make circle all that apply.				
Q4-1	Always wearing mask			
Q4-2	Body temperature and health status grasping			
Q4-3	Disinfection work by teachers			
Q4-4	Social distance			
Q4-5	Activity restriction during class			
Q4-6	Using face shield			
Q4-7	Prohibition or restriction of play and conversation			
Q4-8	Eat without talking /no refills			
Q4-9	Do not let children serve			
